# Fast Measurement and Reconstruction of Large Workpieces with Freeform Surfaces by Combining Local Scanning and Global Position Data

**DOI:** 10.3390/s150614328

**Published:** 2015-06-17

**Authors:** Zhe Chen, Fumin Zhang, Xinghua Qu, Baoqiu Liang

**Affiliations:** State Key Lab of Precision Measuring Technology and Instruments, Tianjin University, Tianjin 300072, China; E-Mails: cz19902013@sina.com (Z.C.); quxinghua@tju.edu.cn (X.Q.); liangbaoqiu1@163.com (B.L.)

**Keywords:** combined measurement, large-scale workpieces with freeform surfaces, multi-sensor data fusion, 3-D reconstruction

## Abstract

In this paper, we propose a new approach for the measurement and reconstruction of large workpieces with freeform surfaces. The system consists of a handheld laser scanning sensor and a position sensor. The laser scanning sensor is used to acquire the surface and geometry information, and the position sensor is utilized to unify the scanning sensors into a global coordinate system. The measurement process includes data collection, multi-sensor data fusion and surface reconstruction. With the multi-sensor data fusion, errors accumulated during the image alignment and registration process are minimized, and the measuring precision is significantly improved. After the dense accurate acquisition of the three-dimensional (3-D) coordinates, the surface is reconstructed using a commercial software piece, based on the Non-Uniform Rational B-Splines (NURBS) surface. The system has been evaluated, both qualitatively and quantitatively, using reference measurements provided by a commercial laser scanning sensor. The method has been applied for the reconstruction of a large gear rim and the accuracy is up to 0.0963 mm. The results prove that this new combined method is promising for measuring and reconstructing the large-scale objects with complex surface geometry. Compared with reported methods of large-scale shape measurement, it owns high freedom in motion, high precision and high measurement speed in a wide measurement range.

## 1. Introduction

Large-scale shape measurement of objects is of great importance in numerous application fields, including cultural heritage protection, film-making, automotive parts, aerospace aircraft, fan blade manufacturing and so on [1,2,3,4]. In most cases, the three-dimensional (3-D) data of an object are desirable because it is necessary for its quantitative analysis. However, as the size increases the measurement accuracy decreases and difficulty increases, especially for irregular shapes.

In the past decades, several acquisition methods have emerged and have been commercialized [5,6,7,8]. Articulated arms, laser trackers, total stations and theodolites can be used to derive high-precision 3-D coordinates, but they are all single point measurement devices. For example, the laser tracker measures the relative position by means of a laser interferometer and a beam-steering mirror using optical encoders. The light source is split into two beams. One beam is used as a reference, and the other beam is reflected back from a retro-reflector. The laser beam hits the retro-reflective target off-center, and it is then reflected back parallel to the incident beam. A sensor measures the displacement [9,10]. Consequently, only a point can be measured every time, therefore the measurement efficiency is too low.

**Figure 1:**
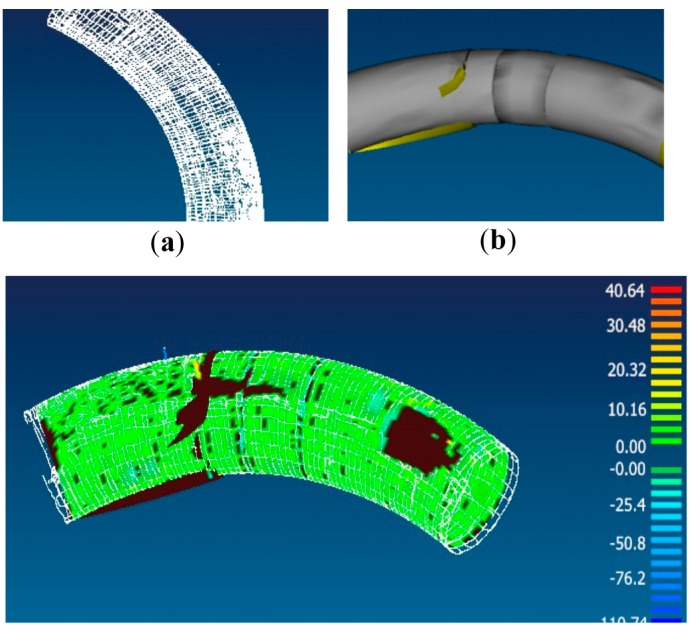
(**a**) The original point cloud; (**b**) Reconstruction surface; (**c**) Error distribution diagram.

To validate that these devices are not suitable for shape measurement, we performed an experiment with a laser tracker to obtain the surface geometry data of a pipe with a diameter of 690 mm, a total length of 1875 mm and a bend curvature radius of 3200 mm. It took 6 h in all to obtain 50 thousands 3-D points, as shown in Figure 1a. However, the surface reconstruction, as shown in Figure 1b, has obvious distortions and the maximum error is up to 110.74 mm, as shown in Figure 1c. These errors are mainly attributed to the sparse data. Therefore, it is difficult for single point devices to be used in the field of surface high-density measurement.

Optical scanning sensors have been proven to be both an accurate and cost-effective alternative to deliver more geometry and appearance information for small objects. They operate on the basis of optical triangulation; that is, a light source (*i.e.*, a single laser spot or a laser stripe) scans an object surface while its reflection is being recorded by one or more CCD cameras. The resulting distance is a function of the CCD inclination angle and the base-length defined between the CCD camera and the laser sensor [11,12]. However, the field of view in a certain station is generally limited to only a few hundred millimeters, so when measuring a large-scale workpiece, the scanning sensor must be placed at different stations around the workpiece. Then the point clouds from the different stations should be registered into a global coordinate system by the corresponding points in the overlapping areas. What’s more, as the size increases, many scans are needed and the number of registrations rises [13]. Thus, optical scanning turns out to be a time consuming and inaccurate process. Consequently it is difficult to obtain the whole morphology and 3-D coordinates of an object with only one certain kind of measuring instrument, so a combination of global position and local measurement is generally used. There have already been some reports on this. Paoli introduced a method based on the integration of a robotic system with an optical scanning sensor for automating the measurement process of built hull yacht shapes [14]. Nevertheless, the measurement range was limited by the length of the robotic arm. Shi proposed a 3-D measurement method by combining an optical scanning sensor with a laser rangefinder. The laser rangefinder was fixed on a guide rail, and the scanning sensor could slide on the guide rail to perform partial section measurement [4]. However, for measuring large-scale workpieces it is necessary to design and manufacture a long guide rail with high precision, so the cost will be greatly increased. To expand the measurement range, Hexagon Metrology, Inc. (Nacka Strand, Sweden), proposed a method of combining a laser tracker and a T-scan, which has a reflector and can be detected by the laser tracker. Nonetheless, the T-scan must always be tracked by the laser tracker, so the scanning speed is constrained [15]. In summary, the existing methods are not the best solutions for large-scale shape measurement, so we propose herein a method involving the combination of a handheld laser scanning sensor and a laser tracker. Our method does not need any mechanical device, and the measurement range is expanded to tens of meters. What’s more, the measurement data is so abundant that the surface shape can be described in detail.

However, only from these points, the surface and geometry information are not available, so we must reconstruct the surface according to these point cloud data. After the surface reconstruction, many studies, such as quality control, non-destructive testing (NDT), product development and numerical simulation including finite element analysis and computational fluid dynamics analysis can all be carried out.

Considerable research has been done for many years on surface models. The Renault engineer Bézier proposed a method to define curves and surfaces with a control polygon to control the curve and surface shape. However, local modification is not supported by the Bezier method, and the continuity of the curve and surface is poor [16]. Gordon and Riesenfeld proposed B-spline curves and surfaces to describe shapes, which overcame the disadvantages of the Bezier methods [17]. However, conic and elementary analytic surfaces cannot be expressed accurately. Syracuse presented the Non-Uniform Rational B-Spline (NURBS) method to offer a common mathematical form for both standard analytical shapes (e.g., conics) and freeform shapes [18]. Recently, NURBS was commonly used in computer graphics for generating and representing curves and surfaces. It offers great flexibility and precision for handling both analytic (surfaces defined by common mathematical formulae) and modeled shapes.

In this work, we construct the large-scale shape measurement system by the combination of local scanning and global positioning. A handheld laser scanning sensor provides precise measurements of high-density points, although, its measurement range is about a few hundred millimeters. Together with the large measurement range of the global position sensor, a laser tracker, the combination of two sensors could compensate each other to guarantee the efficiency, the data density and the precision at the same time. The multiple sensors will generate a mass of data. To transform the data into a global coordinate system, we adopt a multi-sensor data fusion method to decrease the transformation errors. Besides the point cloud data acquisition, the surface reconstruction is important for the shape measurement. In this paper, we propose a method to reconstruct the surface of a large-scale gear rim. It is significant in practical measurement field. Finally, the error analysis and synthesis of the entire system is established.

## 2. Description of the Combined System

### 2.1. System Design

**Figure 2:**
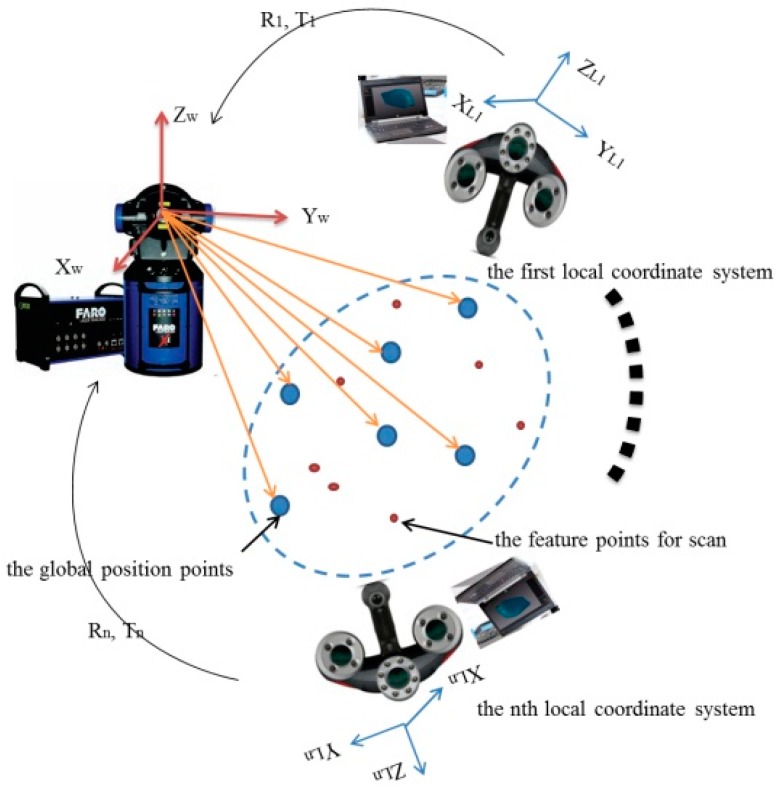
Schematic of the combined system.

As shown in Figure 2, the described system is made up of a handheld laser scanning sensor, a laser tracker, used as a position sensor, and the PC software. Compared with traditional scanning sensors, the handheld laser scanning sensor is more suitable for performing local scanning. Without the limitation of the external mechanical device, the scanner possesses six degrees-of-freedom in motion to eliminate the blind areas, and meanwhile, the scanning process is simplified.

In this paper, we choose a MAXscan device (Creaform, Inc., Québec, QC, Canada), for acquiring the local dense point clouds. It is composed of a class-II laser diode that projects a crosshair over the surface to digitize, and two synchronized cameras with a resolution of 0.1 mm, that capture the image of projections at a high rate. As the relative position of the laser diode and two cameras is calibrated, a triangulation-based algorithm enables the acquisition of the coordinates of the points. Its accuracy is up to 0.05 mm, at a measuring speed of 48,000 scans per second. Therefore, it’s possible to obtain all the surface shape details of complex objects.

**Figure 3:**
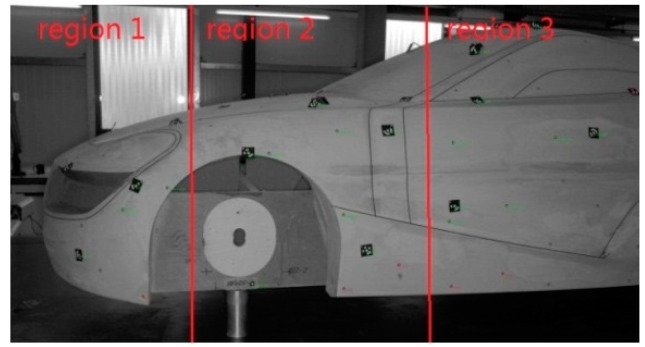
Measuring the distance between region 1 and region 3.

Compared with the total station, the theodolite and the digital photogrammetry system, the measurement accuracy of the laser tracker is higher, and the measurement range is larger. What’s more, the laser tracker is able to link multiple discontinuous partial regions of interest over a large volume into the global coordinate system. For example, the measured volume is so large that it will be separated into several small regions to decrease the accumulated errors in the scanning process. As shown in Figure 3, the bodyshell is divided into three regions. If we are only interested in region 1 and region 3, we just scan region 1 and region 3 and transform them into the global coordinate system with the laser tracker. The method will save a lot of time and improve the measurement efficiency. Therefore we adopt the laser tracker to position a global coordinate system for the handheld laser scanning sensor. A Faro Laser Tracker Xi was chosen for tracking the global position points as reference. Its Absolute Distance Meter (ADM) uncertainty is 10 µm + 1.1 × L µm/m, and the technical information has been verified by the National Metrology Institute of China (NIM CDjx2008-0782).

### 2.2. Multi-Sensor Data Fusion

We assume that the laser tracker’s coordinate system is the global coordinate system, expressed by O-X*_W_*Y*_W_*Z*_W_* and the coordinate system of MAXscan is the local coordinate system O-X*_L_*Y*_L_*Z*_L_*. With the movement of MAXscan, the *i*th local coordinate system is expressed by O-X*_Li_*Y*_Li_*Z*_Li_* (*i* = 1, 2, 3 …, n).

**Figure 4:**
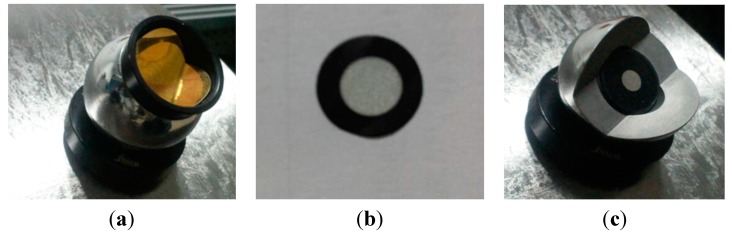
(**a**) TBR for the laser tracker mounted on a nest; (**b**) RRTs pasted on the object; (**c**) RRT pasted on the center of a 1.5-inch tip on the nest to assess the same point with the laser tracker and MAXscan.

When both the sensors are locked onto the same target, integrating the 3-D measurements from different instruments in a global coordinate system is possible. These targets are the reference global position points. However, both instruments have their own specific targets. Wide-angle retro-reflectors known as Tooling Ball Reflectors (TBRs) are used as the probe of the laser tracker as illustrated in Figure 4a, whose centers are considered as the global position points. The retro-reflective targets (RRTs), as shown in Figure 4b, are pasted on the object to facilitate the simultaneous measurement of 3-D surface geometry and estimation of a model of positioning features for tracking, so we must solve the inconsistency. To measure the same physical features, the RRT pasted on the center of the 1.5-inch diameter half-sphere probe tip, which is the commercially available target, is used as shown in Figure 4c. It provides same centerline value as the TBR and it is used as a medium between laser tracker systems to optical scanning systems.

The coordinate of a point in O-X_W_Y_W_Z_W_ is noted by ξ*_W_* = [X*_W_* Y*_W_* Z*_W_*] and that in O-X*_Li_*Y*_Li_*Z*_Li_* is ξ*_L_* = [X*_L_* Y*_L_* Z*_L_*]. The spatial relationship of all the local point clouds relative to the global coordinate system can be achieved by the scale parameter k, the rotation matrix *R* and the translation vector *T*. The coordinate transformation equation is expressed by:

(1) $$\xi_{W}=kR\xi_{L}+T$$

where R = r(ω)r(φ)r(к) = $\left(\begin{array}{ccc} \cos\omega & \sin\omega & 0\\ -\sin\omega & \cos\omega & 0\\ 0 & 0 & 1 \end{array}\right)\left(\begin{array}{ccc} \cos\varphi & 0 & -\sin\varphi \\ 0 & 1 & 0\\ \sin\varphi & 0 & \cos\varphi \end{array}\right)\left(\begin{array}{ccc} 1 & 0 & 0\\ 0 & \cos\alpha & \sin\alpha \\ 0 & -\sin\alpha & \cos\alpha \end{array}\right)$, T = [x_T_ y_T_ z_T_]^T^.

There are seven undetermined parameters in the above matrix equations, so at least three pair-wise registration points, which are non-collinear and able to be observed simultaneously by the two sensors, should be measured to calculate the undetermined parameters. In general, when the rotation angle is large, the error generated by the linear model as Equation (1) cannot be neglected, so we estimate these parameters based on the improved Gauss-Newton optimization approach. Compared to the traditional Gauss-Newton optimization approach method, the improved approach is convergent, no matter what the initial value is. Moreover, the computing speed is improved.

Firstly, we suppose that the error of the linear model is expressed by Equation (2):

(2) $$V=f(X)-\xi_{W}$$

where f(X) is the nonlinear function vector consisted of the unknown parameter vector X = [x_T_ y_T_ z_T_ ω φ к k]^T^.

Based on the traditional solution of the least squares adjustment, the objective function is constructed by:

(3) $$V^{T}PV={\left(f(X)-\xi_{W}\right)}^{T}P(f(X)-\xi_{W})=\min$$

where *P* is weight matrix, and *P* = σ^2^[Var(V)]^−1^, σ^2^ is the variance factor of unit weight.

It means to meet the nonlinear equations:

(4) $${\frac{\partial f(X)}{\partial X}|}_{X=X_{i}}^{T}P(f(X)-\xi_{W})=0$$

Then, Taylor’s expansion in initial value X = [x_T0_ y_T0_ z_T0_ ω_0_ φ_0_ к_0_ k_0_]^T^ is expressed by:

(5) $${\frac{\partial f(X)}{\partial X}|}_{X=X_{0}}^{T}P(f(X_{0})-\xi_{W})+[{\frac{\partial f(X)}{\partial X}|}_{X=X_{0}}^{T}P{\frac{\partial f(X)}{\partial X}|}_{X=X_{0}}+{\left(f(X_{0})-\xi_{W}\right)}^{T}P{\frac{\partial^{2}f(X)}{\partial X_{i}\partial X_{j}}|}_{X=X_{0}}]\delta_{X}=0$$

So:

(6) $$\delta_{X}={\left(B^{T}PB-[l^{T}P][W]\right)}^{-1}B^{T}Pl$$

where $B={\frac{\partial f(X)}{\partial X}|}_{X=X_{0}},l=\xi_{W}-f(X_{0}),W={\frac{\partial^{2}f(X)}{\partial X_{i}\partial X_{j}}|}_{X=X_{0}}$.

Thus the optimal solution is estimated by:

(7) $$\hat{X}=X_{0}+\delta_{X}$$

Finally, in this iterative process, $\hat{X}$ is updated as the initial value until $\delta_{X}$ reaches the threshold value. Threshold value is a tiny number. In this paper, the value is 1 × 10^−5^.

Thus, from Equation (7), we can obtain the rotation matrix R and the translation vector T. However, with the existing of the measurement error, R and T are not accurate enough. As the number of the coordinate transformations rises, errors will accumulate. In this paper, we adopt the multi-sensor data fusion proposed by Nandi to improve the accuracy and decrease the transformation error [19].

For notational ease, M*_i_* (*i* = 1, …, n) is the 3-D coordinate from the ith sensor, and n is the number of the sensors. It usually can be expressed by means of a function of θ*_i_*:

(8) $$M_{i}=f(\theta_{i}+\delta \theta_{i})$$

where $\delta \theta_{i}$ represents the additive noise.

Equation (9) is deduced by Taylor expanding M*_i_* and neglecting the quadratic term:

(9) $$M_{i}=f(\theta_{i})+J_{i}\delta \theta_{i}$$

where J*_i_* is the Jacobian matrix with respect to θ:

(10) $$J_{i}=\frac{\partial f}{\partial \theta_{i}}$$

Assuming a Gaussian distribution for $\delta \theta_{i}$, we get the covariance matrix for the *i*th sensor:

(11) $$V[M_{i}]=E[(M_{i}-\hat{M_{i}}){\left(M_{i}-\hat{M_{i}}\right)}^{T}]=J_{i}V[\delta \theta_{i}]J_{i}^{T}$$

where $\hat{M_{i}}$ is the fused result for M*_i_*:

(12) $$\hat{M_{i}}\text{=}\sum_{i}^{n}W_{i}M_{i}$$

where the weight matrix W*_i_* for the *i*th sensor is determined by the covariance matrix V [M*_i_*]:

(13) $$W_{i}={\left[\sum_{i}^{n}V{\left[M_{i}\right]}^{-1}\right]}^{-1}V{\left[M_{i}\right]}^{-1}$$

Then the covariance matrix V[$\hat{M_{i}}$] is:

(14) $$V[\hat{M_{i}}]={\left[\sum_{i}^{n}V{\left[M_{i}\right]}^{-1}\right]}^{-1}$$

Obviously, from Equation (14), we can obtain that V[$\hat{M_{i}}$]^−1^ > V[Mi]^−1^. Thus, V[$\hat{M_{i}}$] < V[M_i_]. This proves that after the multi-sensor data fusion, the data accuracy is improved.

## 3. Experiments

The proposed system is a portable and high-precision 3-D dense point cloud measurement system. By combining the wide coordinate range control of the laser tracker with the surface scanning measurement of a scanning sensor, the accumulated errors during the scanning process are decreased and the 3-D surface and geometry information of large-scale workpieces with freeform surfaces can be derived quickly and easily. In this section, we perform some experiments to verify the feasibility of the combined measurements. First, a standard value is necessary to assess the metrological performance of the measurement method. Hence, in Section 3.1, we establish the standards for the subsequent experiments. In Section 3.2, small-sized objects are tested separately using only a scanning sensor and the combined method. The combined method we propose in this paper mainly aims at the large-scale measurement. For this reason, large-scale objects are experimented on in Section 3.3. However, fabricating a large-size artifact is difficult, so instead, since one-dimensional length standards are easily acquired, we measure the distances between points and compare the measurement results with the standards. Finally, the differences can be evaluated in terms of the average error.

### 3.1. Standard Establishment

**Figure 5:**
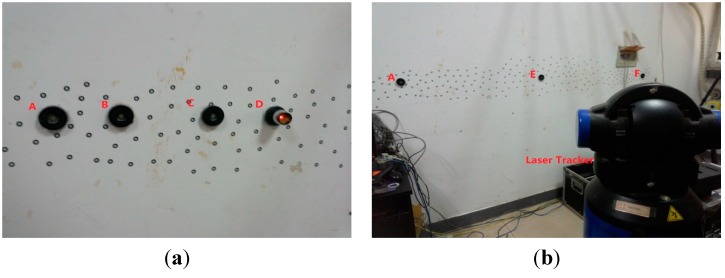
The standard values of the distances between the points measured by the laser tracker. (**a**) The measurement of AB, AC and AD; (**b**) The measurement of AE and AF.

The standard should be of considerably higher accuracy than the measurement method being tested. As the laser tracker’s accuracy is higher than that of the combined method, we measure the coordinate of each point many times to calculate the distance between the points and the average of the distances is used as the standard value. For example, as shown in Figure 5a, four nests are glued to a concrete wall in the experiment and the coordinates are created using nests designed to accept a 1.5-inch TBR. At last, the standard values of AB, AC, and AD are obtained. Meanwhile, in Figure 5b, the standard values of AE and AF are also derived in the same manner. All the standard values are listed in Table 1.

**Table 1:** All the standard values in the experiment.

Object	Standard Value (mm)
**AB**	127.7948
**AC**	329.9191
**AD**	487.4985
**AE**	1015.1636
**AF**	2211.4268

### 3.2. Small Size

In this section, we measure the distance separately using only a scanning sensor, the MAXscan, and the combined system, and then, the validity of the combined method is demonstrated by comparative experiment results.

**Figure 6:**
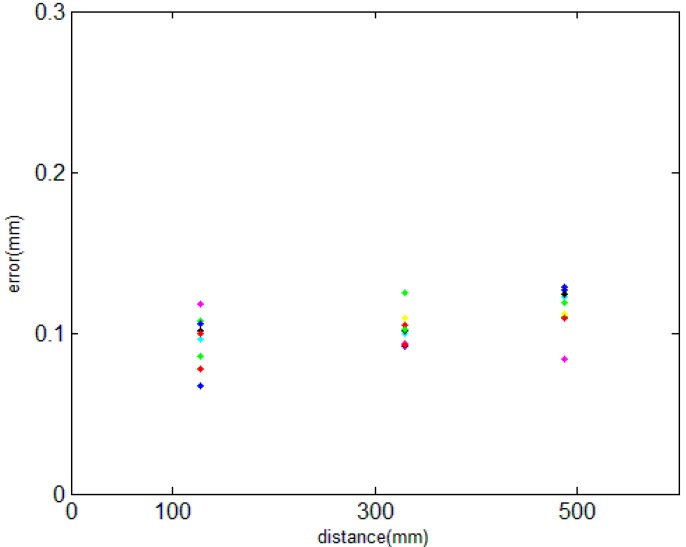
The length of AB, AC and AD measured only by MAXscan.

Firstly, we measure the length of AB, AC and AD only by the MAXscan. The corresponding error can be obtained by comparing it with the standard. This process is repeated 10 times. As seen from Figure 6, as the distance rises, the error grows. The change is not obvious due to the short distance.

**Figure 7:**
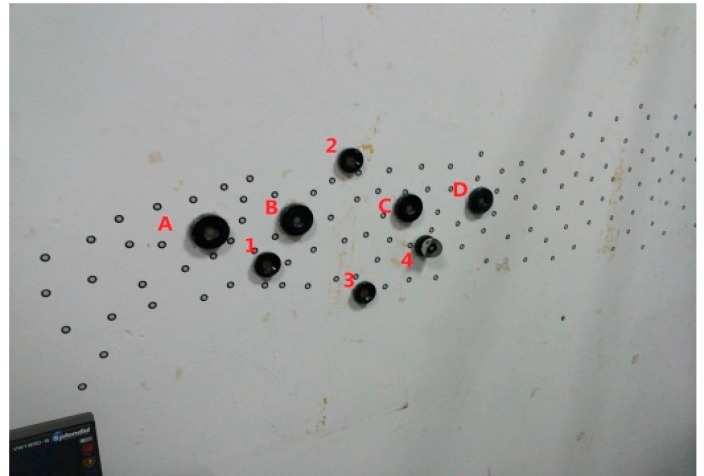
The layout of the reference global position points.

**Figure 8:**
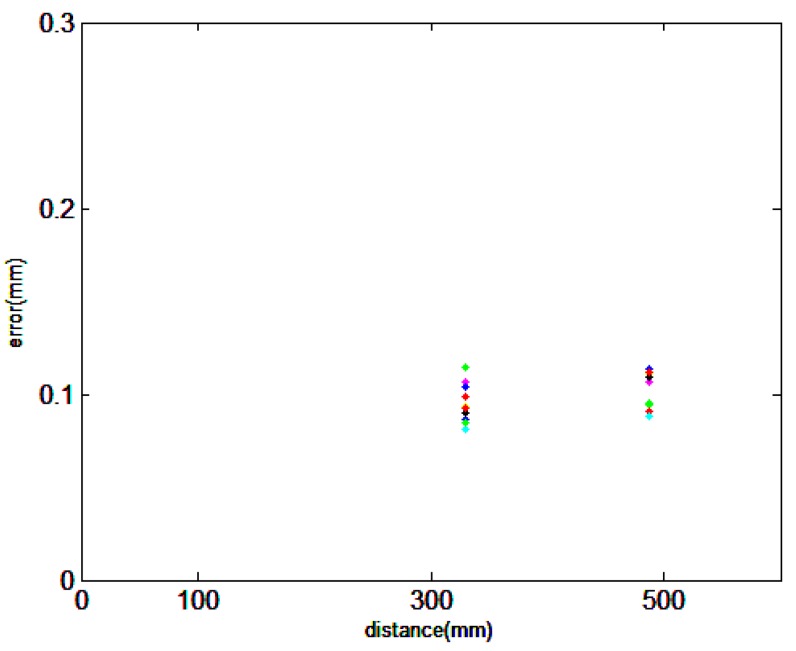
Measurement error of the lengths of AC and AD using the combined method.

**Figure 9:**
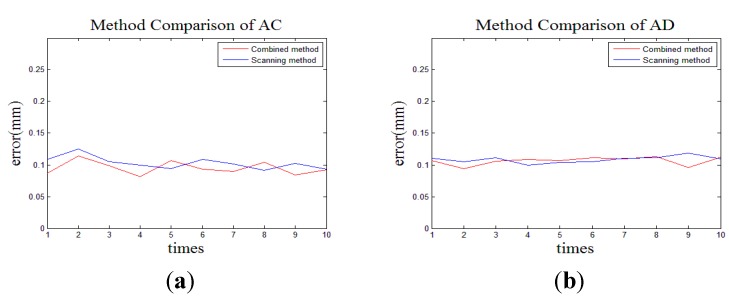
The error comparison by two methods: (**a**) The measured object is AC; (**b**) The measured object is AD.

As the length of AB is too small, we just measure the length of AC and AD by the combined method. Considering that the measurement process is similar, the measurement of AD can be taken as an example to show the measurement process by the combined method. Additional nests are placed for the multi-sensor data fusion between O-X*_W_*Y*_W_*Z*_W_* and O-X*_Li_*Y*_Li_*Z*_Li_*. Then, all the measured volumes are separated to two scanning stations. As shown in Figure 7, points A, 1, 2, 3 and 4 are scanned at station 1, and points D, 1, 2, 3 and 4 are scanned at station 2. Meanwhile, points 1, 2, 3 and 4 are measured by the laser tracker as the reference global position points. Based on the principle of the multi-sensor data fusion, the points from both scanning stations can be transferred into the global coordinate system. Finally, we can calculate the length of AD in the global coordinate system. This process is repeated 10 times, and the results are compared with the standard. The deviations are shown in Figure 8. From Figure 9, through the error comparison by two methods for AC and AD, it is clearly observed that the accuracy is improved by the combined method, demonstrating that the combined method is effective to decrease the accumulated error.

### 3.3. Large Scale

**Figure 10:**
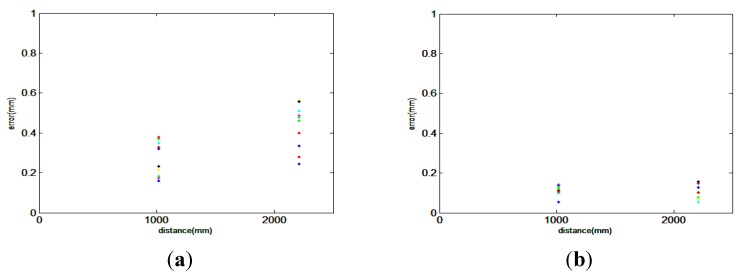
(**a**) Measurement error of AE and AF using the scanner; (**b**) Measurement error of AE and AF using the combined method.

As the combined method is intended for large-scale measurements, it is important to carry out tests on large-scale workpieces. However, experiments on large volumes cannot be performed because of the limited space in the laboratory. The measurement of AE and AF can be taken as examples to evaluate the effect of the combined method. In this case, all the measured volumes are separated into five scanning stations. The measuring process is similar to the one described in Section 3.2. All the results are compared with the standard value, and the deviations are shown in Figure 10. We can conclude that the combined method is a powerful method to decrease the accumulated error.

## 4. Practical Application

**Figure 11:**
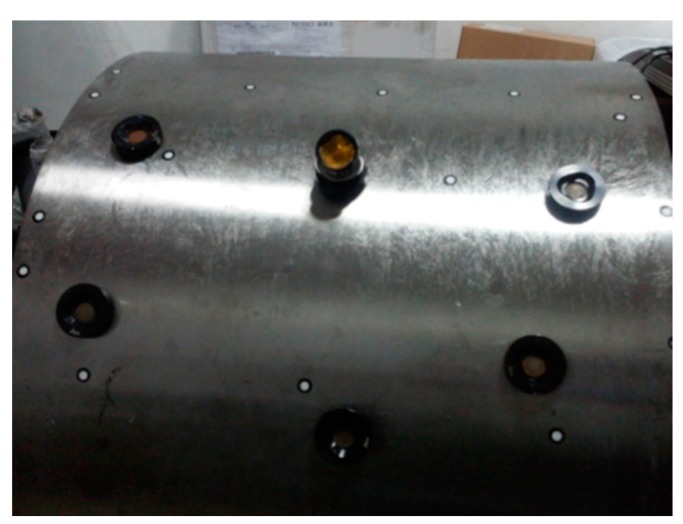
The ring gauge with the reference global position points.

**Figure 12:**
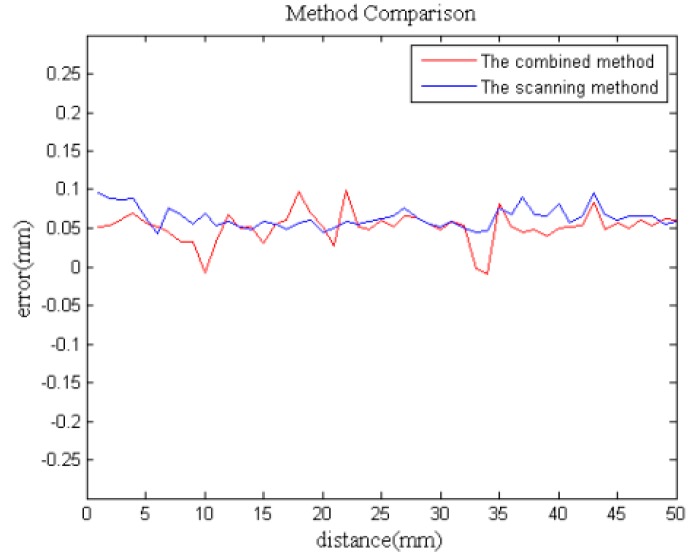
The results of the deviation comparison.

For the purpose of verifying the practicality of the proposed measurement method, a small ring gauge and a large-scale gear rim were chosen as measurement objects. Considering that there is no surface and geometry information to measure the length on the flat concrete wall, we apply this combined method on a ring gauge with the standard length of 500.125 mm, which is measured by the laser tracker, as shown in Figure 11. Similarly, the length is also measured only by MAXscan and the combined system, respectively. The comparative experiment results are shown in Figure 12. It is observed that the length measured by the combined measurement is closer to the standard. It has been successfully verified that combined measurement effectively reduces the accumulated error caused by a large number of image registration and transformation steps and improves the measurement accuracy.

**Figure 13:**
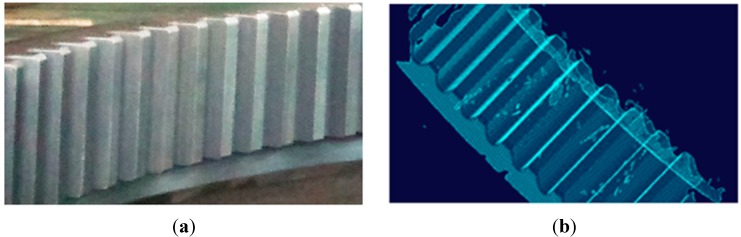
(**a**) A large-scale gear rim; (**b**) The original cloud.

As the ring gauge is small, a large-scale gear rim, as shown in Figure 13a, is measured by the combined method. The gear rim is widely used as a key mechanical part in power stations, dams, offshore platforms and so on. The measurement accuracy during the fabrication and assembly process has a considerable influence on the performance of products.

The gear rim’s radius is 1163 mm, length about 560 mm and width 220 mm. A total of 66,893 points are acquired by the proposed method, as shown in Figure 13b. Then, in order to derive the reconstructed model, a high-accuracy 3-D reconstruction method based on NURBS surface is adopted for data processing and surface reconstruction. In the surface reconstruction, NURBS is better able to control the degree of the curve than the traditional grid modeling approaches, such as Bezier, triangle-Bezier or B-spline, and thus it is commonly used to create a high fidelity model. What’s more, many applications involving CAD/CAM, virtual reality, animation and visualization use models described by NURBS surfaces. Over the last few years, they have become very important in industry, and they are used to represent shapes of automobiles, aeroplanes, ships, mechanical parts, *etc.* [20,21]. Therefore, in this paper, the 3-D solid model is reconstructed based on the NURBS surface, whose mathematical model can be expressed by:

(15) $$P(u,v)=\frac{\sum_{i=0}^{n}\sum_{j=0}^{m}\omega_{ij}N_{ik}(u)N_{jl}(v)P_{ij}}{\sum_{i=0}^{n}\sum_{j=0}^{m}\omega_{ij}N_{ik}(u)N_{jl}(v)},u\in \left[u_{k-1},u_{n+1}\right],v\in \left[v_{l-1},v_{m+1}\right]$$

where P*_ij_* (0 ≤ *i* ≤ n, 0 ≤ *j* ≤ m) is the control points, ω*_ij_* is corresponding weight of P*_ij_*, N*_ik_*(u) and N*_jl_*(v) are the normalized B-spline base functions of order *k* and *l*, respectively, defined over vector U = {u_0_, u_1_, …, u_n_, …, u_n+k_} and V = {v_0_, v_1_, …, v_m_, …, v_m+k_}.

**Figure 14:**
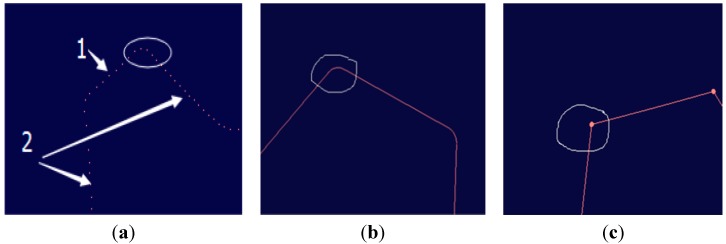
The fitting result comparison: (**a**) The point data; (**b**) The conventional method; (**c**) The proposed method.

We adopt the commercial software package Imageware to reconstruct the surface. The NURBS parameters can be calculated and determined by the software automatically. As the number of points improves, the reconstructed surface becomes a better approximation of the true shape. With the use of the combined measurement method, big curvature surfaces can be scanned densely to provide abundant information and then reconstructed with high fidelity. Meanwhile, the fitting skills also have an impact on the accuracy. As shown in Figure 13, there are fillets on the edge. They are produced by the processing technology to remove burrs, reduce the stress concentration and improve the strength of the gear rim. However, with the existence of the fillets, we cannot extract the structural contours for the gear rim. If the curve is reconstructed just by the software automatically, the reconstruction model will show a round corner as shown in Figure 14b. In this paper, each tooth of the gear is divided into two parts. Part 1 is the addendum circle and part 2 includes the left and right flank of a tooth, as shown in Figure 14a. According to the points in the corresponding part, three lines are reconstructed, respectively. Finally, these lines are extended and trimmed in the intersection, as shown in Figure 14c. This method is not only used in the measurement field, but also applied in practical manufacturing.

**Figure 15:**
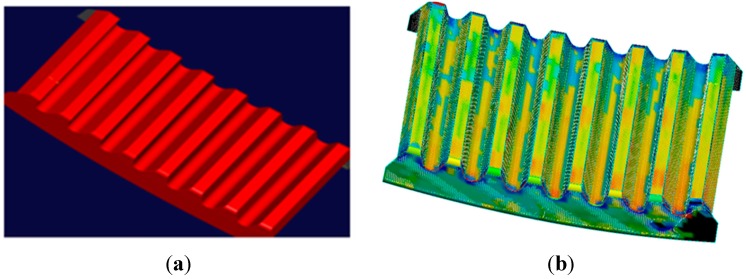
(**a**) The fitting surface; (**b**) Error distribution diagram.

Consequently, the surface model is reconstructed from the point cloud data, as shown in Figure 15a. Furthermore, the error of the reconstructed surface is analyzed. The analysis results are summarized in Table 2. The average Euclidean error in the process of establishing the computer 3-D model of the gear rim is 0.0816 mm. The distribution of the error caused by reconstruction is shown in Figure 15b in which the surface error distribution is in the light yellow band and light blue band, indicating that almost all error is between ±0.1 mm.

**Table 2:** The error evaluation of the surface.

	Mean Error	Max Error	Standard Deviation
Lateral deviation	0.0000	0.0001	0.0000
Negative normal deviation	−0.0821	−0.4300	0.0582
Positive normal deviation	0.0810	0.3477	0.0704
Euclidean error	0.0816	0.4300	0.0647

## 5. Error Analysis and Synthesis

The error analysis and synthesis of the entire system is necessary to guarantee the application of the established system in the industrial field, so the total error model from the measurement entities to the surface model should be established. Error between the measurement entities and the surface model is the cumulative error caused by all aspects of the whole process, which include the measurement error of the laser tracker ∆_W_, the measurement error of the handheld laser scanning sensor ∆_S_, data processing error Δp, the transformation error ∆_T_ and the surface reconstruction error ∆_R_. Assuming all of the errors meet a normal distribution, the total error may be considered as:

(16) $$\text{Δ}=\sqrt{{\text{Δ}}_{\text{W}}^{\text{2}}\text{+}{\text{Δ}}_{\text{S}}^{\text{2}}\text{+}{\text{Δ}}_{\text{P}}^{\text{2}}\text{+}{\text{Δ}}_{\text{T}}^{\text{2}}\text{+}{\text{Δ}}_{\text{R}}^{\text{2}}}$$

The data processing error Δp refers to errors generated from smoothing of noisy data and streamlining of redundant data. Due to only eliminating the obvious noise point of a large area manually during the data processing without smoothing and streamlining, the data processing errors are negligible. After the multi-sensor data fusion, the transformation error is decreased and can be also ignored relative to the measurement error of the laser tracker and the handheld laser scanning sensor [22,23]. The maximum length of the workpiece is 560 mm, and thus the measurement error is:

(17) $${\text{Δ}}_{\text{W}}\text{ = 10 }\mu \text{m + 1.1 }\times \text{0.560 }\mu \text{m = 10.616 }\mu \text{m}$$

(18) $${\text{Δ}}_{\text{S}}=\text{0.05 mm}$$

Meanwhile, as the average Euclidean error is the synthesized error of the surface reconstruction process, the surface reconstruction error ∆_R_ of the gear rim is 0.0816 mm. Thus, according to the Equation (16), the total error is 0.0963 mm.

## 6. Conclusions

An innovative 3-D measurement method involving a combination of local scanning and global positioning has been presented. The method facilitates the precise measurement of large workpieces with freeform surfaces, which have wide applications in the industrial field. The system consists of a handheld laser scanning sensor and a laser tracker. Based on the principle of multi-sensor data fusion, all the local point clouds can be precisely integrated into the same global coordinate system established by the position sensor. Therefore, this system solves the problem that as measured range increases, the scanning accuracy diminishes due to the increased number of registrations. Besides, the measurement range is expanded to tens of meters. We perform experiments in the lab to validate that the proposed method eliminates the accumulated registration errors that exist specially in the large workpiece measurement process and improves the measurement accuracy. In these experiments, different measurement ranges are selected. After that, the combined method is applied to a small ring gauge and a large-scale gear rim to demonstrate the practicability in practical applications. We also propose the reconstruction method of the gear rim based on the NURBS surface method. Finally, we establish the total error model to analyze and synthesize the errors of the entire system. Although the measurement range is up to tens of meters, the synthesized error of the measurement and reconstruction is less than 0.0963 mm. The proposed method is also cheaper compared with other existing systems. Nevertheless, the data processing and the surface reconstruction using Imageware are complicated, therefore, future work will seek to develop special software to simplify the data processing and reconstruction processes.
